# H3K27Me3 abundance increases fibrogenesis during endothelial-to-mesenchymal transition via the silencing of microRNA-29c

**DOI:** 10.3389/fcvm.2024.1373279

**Published:** 2024-05-07

**Authors:** Jolien Fledderus, Linda Brouwer, Timara Kuiper, Martin C. Harmsen, Guido Krenning

**Affiliations:** ^1^ Laboratory for Cardiovascular Regenerative Medicine, Medical Biology Section, Department Pathology and Medical Biology, University Medical Center Groningen, University of Groningen, Groningen, Netherlands; ^2^ Division Experimental Pharmacology, Department Clinical Pharmacy and Pharmacology, University Medical Center Groningen, University of Groningen, Groningen, Netherlands

**Keywords:** EZH2, H3K27Me3, EndMT, miR-29c, fibrogenesis

## Abstract

**Objective:**

Endothelial-to-mesenchymal transition (EndMT) is a transdifferentiation process in which endothelial cells (ECs) adopt a mesenchymal-like phenotype. Over the past few years, it became clear that EndMT can contribute to several cardiovascular pathologies. However, the molecular pathways underlying the development of EndMT remain incompletely understood. Since the epigenetic enzyme Enhancer of Zeste Homolog 2 (EZH2) and its concomitant mark H3K27Me3 have been shown to be elevated in many cardiovascular diseases that associate with EndMT, we hypothesized that H3K27Me3 is a determinant for the susceptibility of EndMT.

**Methods:**

To study the association between H3K27Me3 and EndMT, a knockdown model of EZH2 in human endothelial cells (HUVEC) was utilized to reduce H3K27Me3 abundance, followed by induction of EndMT using TGFβ1. The expression of molecular markers of EndMT and fibrogenesis were analysed.

**Results:**

In cultured HUVECs, a reduction of H3K27Me3 abundance facilitates EndMT but mitigates fibrogenesis as shown by a decreased expression of collagen I and III. In HUVEC, H3K27Me3 abundance directly affects the expression of miR29c, a collagen-targeting miRNA. Additionally, knockdown of miR-29c in HUVEC with low H3K27Me3 abundance partly restored the expression of collagen I and III. Expectedly, in rats with perivascular fibrosis an increased abundance of H3K27Me3 associated with a decreased expression of miR-29c.

**Conclusion:**

our data shows that endothelial fibrogenesis underlies an epigenetic regulatory pathway and we demonstrate that a decreased abundance of H3K27Me3 in ECs blunts fibrogenesis in part in a miR-29c dependent manner. Therefore, a reduction of H3K27Me3 could serve as a novel therapeutical strategy to mitigate fibrogenesis and may prove to be beneficial in fibrogenic diseases including atherosclerosis, cardiac fibrosis, and PAH.

## 1 Introduction

Endothelial-to-mesenchymal transition (EndMT) is a transdifferentiation process in which endothelial cells (ECs) adopt a fibroproliferative mesenchymal-like phenotype. Transforming growth factor β (TGFβ) plays a pivotal role in EndMT induction. Canonical TGFβ signalling causes induction of mothers against decapentaplegic homolog 2/3 (Smad2/3) which leads to repression of endothelial genes and induction of mesenchymal gene expression whereas, non-canonically TGFβ can induce EndMT via the p38 mitogen-activated protein kinase (MAPK) signalling pathway leading to the induction of the mesenchymal transcription factor Snail (1, 2). Other signalling mechanisms such as increased reactive oxygen species (ROS) can induce EndMT by inducing endogenous TGFβ expression via the nuclear factor kappa-light-chain-enhancer of activated B cells (NF-κB) pathway (3, 4). During EndMT, ECs lose expression of their typical markers such as vascular endothelial cadherin (VE-cadherin) and platelet endothelial cell adhesion molecule (PECAM-1 or CD31), while the expression of mesenchymal-like markers such as smooth muscle protein 22-alpha (SM22α) and alpha-smooth muscle actin (αSMA) is induced. Additionally, ECs adopt a mesenchymal phenotype including a spindle-shaped elongated morphology and acquisition of cellular motility and invasive and contractile properties (5, 6). These phenotypical changes coincide with functional changes as indicated by reduced angiogenic potential, reduced nitrogen oxide (NO) production and reduced anti-thrombogenic behaviour, whereas mesenchymal traits such as contractile behaviour, migratory potential, and deposition of extracellular matrix proteins such as collagens is gained (5, 7–9).

Although, over the past years it became evident that EndMT contributes to several cardiovascular pathologies in adult life, including pulmonary arterial hypertension (PAH), cardiac fibrosis, and atherosclerosis (7, 9–13), the molecular pathways underlying EndMT remain incompletely understood. For example, the increased deposition of fibrillar collagens during EndMT and its association with the occurrence of fibroproliferative disorder suggests that EndMT may be a critical factor in pathogenesis. Indeed, the selective inhibition of EndMT in advanced atherosclerotic plaques not only halts atherosclerosis progression but also reduced the number of readily formed lesions (14).

The phenotypic changes during EndMT are essentially a reprogramming of the transcriptional profile. While much pertains to canonical transcription factor-regulated gene expression, EndMT is tightly regulated by epigenetic rewiring too. Epigenetics refers to the regulation of gene expression at the chromatin level by shaping the accessibility of gene promoters to transcription factors. Changes in the epigenome have increasingly been linked to EndMT [reviewed in (15)] and the development and progression of cardiovascular disease (CVD) (16, 17). For example, increased expression of the histone methyltransferase Enhancer of Zeste Homolog 2 (EZH2) is associated with EC dysfunction. EZH2 is the catalytic subunit of the Polycomb Repressive Complex 2 and tri-methylates lysine 27 of histone 3 (H3K27Me3) thereby causing transcriptional repression of corresponding gene. EZH2 and its concomitant repressive histone mark H3K27Me3 play a major role in endothelial homeostasis, through the regulation of genes involved in cell cycle, cell communication and cell adhesion (18–20). Low expression levels of EZH2 and the correspondingly low abundance of H3K27Me3 in ECs promotes quiescence, thereby promoting endothelial homeostasis (18, 21). Interestingly, elevated expression levels of EZH2 and abundance of H3K27Me3 associate with several CVDs, including PAH, cardiac fibrosis, and atherosclerosis (21–23). More specifically, in experimental rodent models of PAH, EZH2 expression is associated with reactive oxygen species (ROS) production and an increase in right ventricular systolic pressure and right ventricular hypertrophy (24, 25). Concurrently, pharmacological inhibition of EZH2 activity ameliorates PAH by mitigating ROS in these models (25). As EZH2 and its concomitant mark H3K27Me3 are increased during CVDs that associate with EndMT, we hypothesized that the increase in H3K27Me3 may occur in the endothelium and is a determinant of EndMT susceptibility. Therefore, in this study we investigated the association between H3K27Me3 abundance and EndMT, using a knockdown model of EZH2 to reduce H3K27Me3 abundance and analysed the expression of molecular markers of EndMT and fibrogenesis. Additionally, our findings were translated to CVD pathology, using an EndMT-prone model of pulmonary arterial hypertension in rats.

## 2 Materials and methods

### 2.1 Animals and procedures

All animal experiments were reviewed and approved by the Animal Experiments Committee of the Groningen University Medical Center (#AVD10500015129) and performed in accordance with the recommendations for animal experiments issued by the European Commission directive 2010\63. Pulmonary arterial hypertension (PAH) induction in Wistar rats (7–9 weeks old; Harlan, Horst, Netherlands) was performed by a single intraperitoneal injection of monocrotaline (60 mg/kg) followed by placement of an aortocaval (AC) shunt one week later as described previously (26, 27). 4 weeks post AC-shunt placement, rats were euthanized by continuous inhalation of 3%–5% isoflurane in air for the duration of the procedure. Hemodynamic examination was performed under continuous inhalation of 3%–5% isoflurane in air for the duration of the procedure, prior to euthanisation using the closed chest technique as described previously (28). A fluid-filled pressure catheter was inserted into the right internal jugular vein and guided to the pulmonary artery while monitoring pressure waveform using a bedside monitor. Right ventricular systolic and diastolic pressure, pulmonary arterial pressure, and pulmonary Wedge pressure were measured. Mean pulmonary arterial pressure (mPAP) was calculated as follows: mPAP(mmHg)=(2/3dPAP+1/3sPAP), in which dPAP and sPAP are diastolic and systolic pulmonary artery pressure, respectively. Total pulmonary vascular resistance was estimated as: $\mathrm{PVR}(\mathrm{mmHg}\cdot \mathrm{ml}\cdot \mathrm{mi}n^{-1})=\frac{\left(\mathrm{mPAP}-\mathrm{PCWP}\right)}{\mathrm{CO}}$, in which mPAP and CO are pulmonary capillary wedge pressure and cardiac output, respectively. Rats were euthanized under 3%–5% isoflurane anaesthesia by exsanguination. Hearts were harvested and processed for further analyses. The hearts were weighed and dissected into the left atrium, left ventricle, right atrium, right ventricle, and septum, which were all weighted individually. The right ventricle was cut in half and one half was fixed in 3.6% formalin and embedded in paraffin, the other half was snap frozen using liquid nitrogen.

### 2.2 Cell culture and stimulation

Human umbilical vein endothelial cells (HUVEC; Lonza, Walkersville, MD) were cultured on 1% gelatin-coated culture plastics in endothelial cell medium consisting of RPMI 1640 (Lonza, Verviers, Belgium) supplemented with 20% Fetal Bovine Serum (FBS; Sigma, St. Louis, MO, USA), 50 µg/ml Endothelial Cell Growth Factors [ECGF; own isolate according to Burgess et al. (29)], 2 mm L-glutamine (Lonza, Basel, Switzerland), 1% penicillin/streptomycin (Gibco, Waltham, MA, USA) and 5 U/ml heparin (Leo Pharma, The Netherlands). Confluent HUVEC were dissociated with Trypsin EDTA in PBS (Gibco). HUVEC were used between passage 3 and 7. EndMT was initiated by culturing HUVEC in RPMI 1640 supplemented with 20% FBS, 1% penicillin/streptomycin, 2 mm L-glutamine, and 5 U/ml heparin and 10 ng/ml TGFβ1 (Peprotech, NJ, USA) for 72 h. Human Embryonic Kidney (HEK293T) cells were cultured in DMEM (Lonza, Basel, Switzerland) supplemented with, 10% FBS, 2 mm L-glutamine, and 1% penicillin/streptomycin.

### 2.3 Lentiviral transduction

HEK293T cells were transfected in 75 cm^2^ plates in a reaction volume of 10 ml with 0.38 µg/ml pLKO.1-shEZH2 or 0.38 µg/ml pLKO.1-shSCR, 0.09 µg/ml pVSVG (envelope plasmid) and 0.38 µg/ml pCMV-R8.91 (gag-pol 2nd generation packaging plasmid) using Endofectin™ Lenti transfection reagent (GeneCopoeia, Rockville, MD, USA). 24 h post-transfection, HEK293T cells were placed on endothelial cell medium. 48- and 72-hours post-transfection, viral supernatants were collected, centrifuged at 500xg, and filtered through 0.45 µm filters. Viral supernatants were supplemented with 6 mg/ml polybrene (Sigma) and added to 70% confluent HUVEC (P3 up to P7) for two consecutive rounds of 24 h exposure. Transduced HUVEC were passaged once, and transduced cells were selected with 4 µg/ml puromycin O/N after which they were used for downstream stimulation or analysis.

### 2.4 Anti-miR transfection

HUVEC were pre-incubated with reduced serum medium OptiMEM (Gibco). Cells were transfected with 50 µM hsa-miR-29c-3p miRCURY LNA miRNA inhibitor (Qiagen, Hilden, Germany) using Lipofectamine 2000 reagent (Invitrogen, Waltham, MA, USA) according to manufacturer's protocol. Cells were incubated with the small interfering RNA (siRNA)/Lipofectamine mix for 4 h at 37°C and 5% CO_2_ after which transfection medium was replaced by endothelial cell medium. The next day, HUVEC were used for downstream stimulation or analysis.

### 2.5 Gene expression analysis

RNA was isolated using in TriZOL reagent (Ambion, Austin, TX, USA) in accordance with manufacturer's protocol. RNA concentration and purity were determined using a Nanodrop 1,000 spectrophotometer (Thermo Scientific, Waltham, MA, USA). cDNA synthesis was performed using RevertAid^TM^ First Strand cDNA Synthesis Kit (Thermo Scientific), according to manufacturer's protocol. 5 ng of total cDNA for gene transcript analysis was amplified. For analysis of microRNA transcripts, the ABI Taqman microRNA reverse transcription kit (Thermo Scientific) was used according to manufacturer's instructions using microRNA specific stem-loop primers (Table 1). 10 ng of total cDNA for microRNA expression analysis was amplified. For all transcript analyses, cDNA was amplified in duplicate on the Viia7 Real-Time PCR System (Thermo Fisher) in a reaction with 0.6 µM primers (gene transcript analysis, Table 2) or 0.5 µM primers (microRNA transcript analysis, Table 2) using SYBR Green chemistry (Roche Diagnostics GmbH, Mannheim, Germany). Data was analysed using the Viia7 software (Thermo Scientific). Cycle threshold (Ct) values for individual reactions were determined and normalized against GAPDH or ACTB (gene transcript analysis) or RNU6 (microRNA transcript analysis). Data is shown as fold change compared to control and was calculated using the 2^−*ΔΔ*Ct^ method.

**Table 1:** Primer sequences stem-loop primers.

Gene	Stem-loop sequences
RNU6	GTCGTATCCAGTGCAGGGTCCGAGGTATTCGCACTGGATACGACAAAAATATGG
miR-29a	GTCGTATCCAGTGCAGGGTCCGAGGTATTCGCACTGGATACGACTAACCGAT
miR-29b	GTCGTATCCAGTGCAGGGTCCGAGGTATTCGCACTGGATACGACAACACTGA
miR-29c	GTCGTATCCAGTGCAGGGTCCGAGGTATTCGCACTGGATACGACTAACCGAT
let-7b	GTCGTATCCAGTGCAGGGTCCGAGGTATTCGCACTGGATACGACAACCACAC
let-7d	GTCGTATCCAGTGCAGGGTCCGAGGTATTCGCACTGGATACGACAGAAAGGC
let7-g	GTCGTATCCAGTGCAGGGTCCGAGGTATTCGCACTGGATACGACAACTGTAC
miR-98	GTCGTATCCAGTGCAGGGTCCGAGGTATTCGCACTGGATACGACAACAATAC

**Table 2:** Primer sequences.

Gene	Forward	Reverse
microRNA analysis		
RNU6	TGCGGCTGCGCAAGGATGA	CCAGTGCAGGGTCCGAGGTCCG
miR-29c	TGCGGTAGCACCATCTGAA	CCAGTGCAGGGTCCGAGGTCCG
miR-29b	TGCGGTAGCACCATTTGAAA	CCAGTGCAGGGTCCGAGGTCCG
miR-29c	TGCGGTAGCACCATTTGAA	CCAGTGCAGGGTCCGAGGTCCG
let-7b	TGCGGTGAGGTAGTAGGTT	CCAGTGCAGGGTCCGAGGTCCG
let-7d	TGCGGCTATACGACCTGCT	CCAGTGCAGGGTCCGAGGTCCG
let7-g	TGCGGTGAGGTAGTAGTTT	CCAGTGCAGGGTCCGAGGTCCG
miR-98	TGCGGTGAGGTAGTAAGTT	CCAGTGCAGGGTCCGAGGTCCG
Gene expression analysis		
GAPDH	AGCCACATCGCTCAGACAC	GCCCAATACGACCAAATCC
ACTB	CCAACCGCGAGAAGATGA	CCAGAGGCGTACAGGGATAG
EZH2	GCGAAGGATACAGCCTGTGCACA	AATCCAAGTCACTGGTCACCGAAC
CDH5	GTTCACCTTCTGCGAGGATA	GTAGCTGGTGGTGTCCATCT
PECAM1	GCAACACAGTCCAGATAGTCGT	GACCTCAAACTGGGCATCAT
NOS3	AGGAACCTGTGTGACCCTCA	TATCCAGGTCCATGCAGACA
TEK	CCCCTATGGGTGTTCCTGT	GCTTACAATCTGGCCCGTAA
TGLN	CTGAGGACTATGGGGTCATC	TAGTGCCCATCATTCTTGGT
CNN1	CCAACCATACACAGGTGCAG	TCACCTTGTTTCCTTTCGTCTT
ACTA2	CTGTTCCAGCCATCCTTCAT	TCATGATGCTGTTGTAGGTGGT
FSP1	CGCTTCTTCTTTCTTGGTTTGA	CGAGTACTTGTGGAAGGTGGA
COL1A1	GGGATTCCCTGGACCTAAAG	GGAACACCTCGCTCTCCA
COL3A1	CTGGACCCCAGGGTCTTC	CATCTGATCCAGGGTTTCCA
ChIP analysis		
miR-29c	AGTTGGCATGAGGCTTCG	ACACAGGCTGACCGATTTCT
miR-98	TCTGTCCCCTTCCATGTCTC	TCCCTGGTGTGTGGCATATT

### 2.6 Chromatin immunoprecipitation (ChIP)

Cells were trypsinized, pelleted and the chromatin was crosslinked with 1% formaldehyde (Sigma) for 8 min. Crosslinking was stopped by the addition of 125 mm glycine (104201, Merck, Kenilworth, NJ, USA). Cell pellets were lysed on ice with SDS lysis buffer (1% SDS, 10 mm EDTA, 50 mm Tris-HCl pH 8.0) supplemented with freshly added 100 mm proteinase inhibitor cocktail (P8340, Sigma) for 15 min. The chromatin was fragmented using the Biorupter (Diagenode, Seraing, Belgium) by applying 5 cycles (30″ ON/OFF) and cleared by centrifugation. Chromatin was diluted 10 times with RIPA buffer (0.1% SDS, 0.1% Na-deoxycholate, 1% Triton-X-100, 1 mm EDTA, 0.5 mm EGTA, 10 mm Tris-HCl, 140 mm NaCl) supplemented with freshly added 100 mm proteinase inhibitor cocktail (Sigma). 40 µl Protein A Dynabeads (#10002D, Invitrogen) were coated with 4 µg H3K27Me3 (07-449, Merck Millipore, Burlington, MA, USA) or IgG control (ab46540, Abcam) and added to the chromatin followed by incubation at 4°C O/N while rotating. The beads were collected using a magnet and washed 3 times with ice-cold PBS, the remaining complexes were eluted with elution buffer (100 mm NaHCO_3_, 1% SDS) for 15 min. at RT. Eluted samples were supplemented with 5M NaCl and RNAse (EN0531, Thermo Scientific) and incubated for 4 h at 62°C to reverse crosslinking. 2 µl proteinase K (03115828001, Roche) was added and incubated for 1 h at 62°C. DNA fragments were purified with a QIAquick PCR purification kit (Qiagen) according to manufacturer's protocol and quantified by qRT-PCR using primers for miR-29c-3p and miR-98 (Table 2). Data is shown as fold enrichment over control IgG values.

### 2.7 Immunoblotting

Cells were harvested in RIPA buffer (Thermo Scientific) supplemented with 1% v/v protease inhibitor cocktail (#P8340, Sigma) and 1% v/v phosphatase inhibitor cocktail (#1861277, Sigma). Samples were sonicated and centrifuged, and protein concentration was measured with a DC protein assay (BioRad, Hercules, CA, USA). Equal amounts of protein were separated by electrophoresis on polyacrylamide gels followed by protein blotting onto nitrocellulose membranes using the semi-dry Transblot Turbo system (BioRad). Membranes were blocked in 5% Elk milk powder (FrieslandCampina, Amersfoort, the Netherlands) in TBS-T at RT for 1 h, and incubated with antibodies against GAPDH (1:2,000, CS-5274S, Cell Signaling, Danvers, MA, USA), EZH2 (1:1000, CS-5246S, Cell Signaling), VE-cadherin (1:1,000, CS-2500, Cell Signaling), eNOS (1:1,000, bs-610296, BD Bioscience, Franklin Lakes, NJ, USA), SM22α (1:1,000, ab14106, Abcam, Cambridge, England) or αSMA (1:1,000, ab124964 Abcam) at 4°C O/N. Membranes were washed twice in TBS-Tween (0.1%), once in AP detection buffer (100 mm NaCl, 100 mm Tris-Base, 50 mm MgCl_2_, pH 9.5) and developed using AP detection buffer supplemented with 5-bromo-4-chloro-3′-indolyphosphatae p-toluidine salt (BCIP, 165 µg/ml) and nitro blue tetrazolium chloride (NBT, 330 µg/ml). Densitometry analysis was performed using Totallab 120 (Nonlinear Dynamics, Newcastle upon Tyne, England). Data is shown as fold change compared to control.

### 2.8 Immunofluorescent staining of cells

Cells were trypsinized and counted. Cytospins of the samples were prepared using the Cytospin 4 centrifuge (Thermo Scientific) using a cell suspension of 25,000 or 50,000 cells per 100 µl endothelial cell medium. Cytospins were made by spinning 5 min. at 550 rpm with low acceleration followed by air drying for 1 h at RT and stored at −20°C. Prior to staining the slides were thawed under a fan for 10 min. after which they were fixed with 2% paraformaldehyde in PBS for 30 min. at RT. For analysis of intracellular proteins, cells were permeabilized with 0.5% Triton-X-100 solution for 15 min at RT. Blocking of non-specific antibody binding was performed using 5% donkey serum in PBS for 10 min. at RT. Samples were incubated with antibodies against H3K27Me3 (1:200, 07-449, Merck Millipore) in PBS supplemented with 5% donkey serum at 4°C O/N. Samples were washed 3 times with 0.05% Tween-20 in PBS and incubated with Alexa Fluor® 594-conjugated antibodies (1:500, #A21207, Invitrogen) against rabbit IgG or Alexa Fluor® 647 conjugated antibodies (1:500, #A31571, Invitrogen) against mouse IgG in DAPI/PBS with 2% human serum for 1 h at RT. Samples were washed 3 times with 0.05% Tween-20 in PBS and spots were fixed with Citifluor covered with a coverslip and stored at 4°C until analysis. Samples were imaged on the EVOS FL (Life Technologies), and analysis was performed using ImageJ software (National Institutes of Health, Bethesda, MD, USA).

### 2.9 Histochemistry

Perivascular fibrosis was scored after picrosirius red staining (PSR) and counterstaining with Weigert's haematoxylin (both Sigma-Aldrich, St. Louis, MO) following manufacturer's instruction. Samples were imaged on a NanoZoomer digital slide scanner (Hamamatsu Photonics, Shizuoka, Japan. For the quantification of perivascular fibrosis, 12–15 arteries per animal were scored positive (PSR^+^) or negative (PSR^−^) for PSR staining and the percentage of PSR^+^ arteries per animal was calculated (PSR^+^ arteries/total arteries × 100). Perivascular fibrosis was quantified using Aperio ImageScope (Leica Biosystems, Nussloch, Germany). For CD31/SM22α and CD31/H3K27Me3 staining on formalin-fixed, paraffin-embedded sections, prior to immunohistochemistry, deparaffinization was performed by placing sections in xylene I (15 min), xylene II (10 min), 100% ethanol (10 min), 96% ethanol (3 min), 70% ethanol (3 min) and demi water (3 min). Heat-induced antigen retrieval was performed with 0.1 M Tris/HCl (pH 9.0) at 80°C for 16 h. Blocking of non-specific antibody binding was performed using 1% BSA and 5% donkey serum in PBS for 30 min. at RT. Sections were incubated with antibodies against H3K27Me3 (1:200, 07-449, Merck Millipore) or SM22α (1:200, ab14106, Abcam) for 1 h at RT. Sections were washed 3 times followed by incubation with Alexa Fluor® 555-conjugated antibodies (1:500 #A31572, Invitrogen) against rabbit IgG 30 min at RT. Sections were washed 3 times, followed by incubation of the second primary antibody: CD31 (1:200, #AF3628, R&D Systems, Minneapolis, MN, USA) for 1 h at RT. After 3 washing steps, sections were incubated with the second secondary antibody: Alexa Fluor® 647 conjugated antibodies (1:500 #A21447, Invitrogen) against goat IgG in DAPI /PBS for 30 min at RT. Sections were washed 3 times and fixed with Citifluor covered with a coverslip and stored at 4°C until analysis. Sections were imaged on the TissueFAXS (TissueGnostics, Vienna, Austria) in combination with Zeiss AxioObserver Z1 microscope. Image analysis was performed using TissueQuest fluorescence (TissueGnostics) software.

### 2.10 Quantification of collagen I and III protein expression

Collagen production by endothelial cells was performed as described elsewhere (30). In short, HUVEC (control, shSCR and shEZH2) were cultured in 96-wells plates at a density of 50,000 cells/cm^2^ and cultured for 96 h with fresh culture medium every 24 h. Where appropriate, HUVEC were stimulated with 10 ng/ml TGFβ1 for 96 h. Cells were gently lysed in extraction buffer (0.5% v/v Triton X-100, 20 mm NH_4_OH in PBS) and the remaining matrix rinsed twice in PBS and fixed using 4% paraformaldehyde at RT for 20 min. Samples were blocked in 5% BSA and subsequently incubated with antibodies against collagen type 1 (1:1000, #ab34710, Abcam plc, UK) and collagen type 3 (1:1,000, #ab7778, Abcam plc, UK) at RT for 1 h. After three washes, samples were visualized using AF555-conjugated secondary antibodies (1:2000, #A31572, ThermoFisher) and recorded on a Varioscan spectrofluorometer (Thermo Scientific, Waltham, MA, USA) and plotted against standards of collagen. All samples were normalized against DNA content as measured in the cell lysates using FluoReporter Blue (#F2962, ThermoFisher Scientific) according to manufacturer's instructions. DNA standards were prepared in extraction buffer.

### 2.11 Statistical analysis

Statistical analysis was performed using GraphPad Prism software version 8 or 9 (San Diego, CA, USA) with an unpaired *t*-test when comparing the means of two groups or a 1-way ANOVA when comparing the means of more than 2 groups. Outliers within a group were detected with a ROUT outlier test (Q = 1%) and significant outliers were excluded from the analysis. Graphs and tables depict the mean with standard deviation. The number of independent experiments or animals used are indicated in the figure legend. *P*-values <0.05 were considered statistically significant.

## 3 Results

### 3.1 Reduction of H3K27Me3 abundance does not impede EndMT but mitigates fibrogenesis

**Figure 1:**
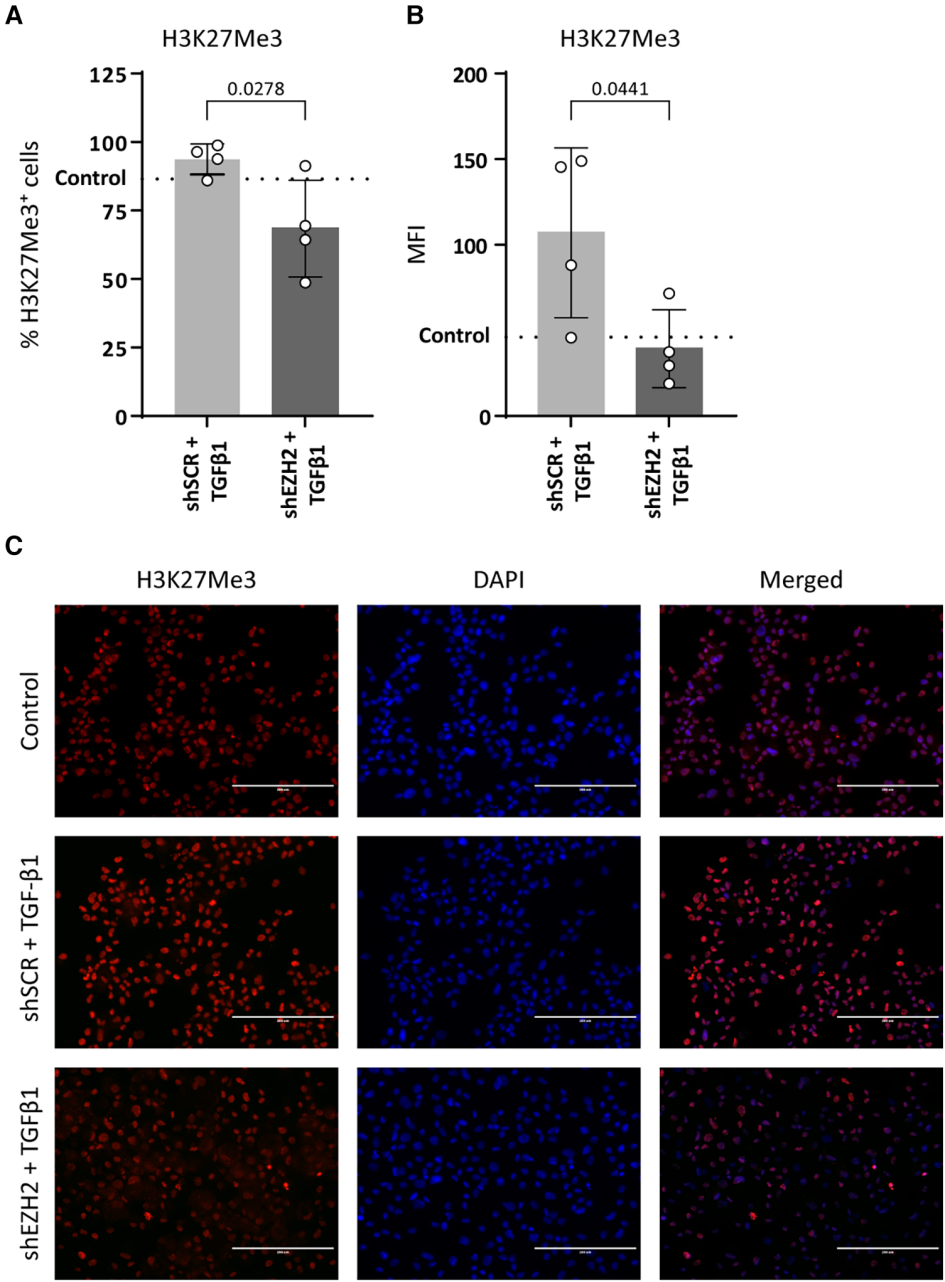
H3K27Me3 expression is reduced in EZH2 deficient HUVECs. HUVECs were stably transduced with a short hairpin construct directed against EZH2 (shEZH2) or with a non-targeting control (shSCR) and stimulated with 10 ng/ml TGFβ1 for 72 h. Quantification of H3K27Me3 expression is shown as the (**A**) percentage of H3K27Me3 positive cells and (**B**) mean fluorescent intensity (MFI) over total amount of cells (*n* = 4). Dotted lines represent average shSCR control value. (**C**) Representative immunofluorescent images of shSCR control, shSCR + TGFβ1 and shEZH2 + TGFβ1 treated HUVECs stained with H3K27Me3 (red), Nuclei are stained with DAPI (blue). Scale bars represent 200 µm. Data presented as mean ± SD, *P*-values from one-way ANOVA with Sidak's multiple comparison tests.

To investigate the correlation between H3K27Me3 abundance and EndMT, we depleted its producing methyltransferase EZH2 in HUVEC using shRNA. shRNA targeted against EZH2 (shEZH2) decreased its expression at the gene and protein level by 3.3-fold and 13.9-fold, respectively, when compared to HUVEC that were transduced with a scrambled control sequence (shSCR, Supplementary Figure S1). Concurrently, the amount of H3K27Me3^+^ cells and the mean fluorescent intensity (MFY) was decreased in EZH2 deficient HUVECs (1.4-fold and 2.7-fold, respectively) when compared to shSCR (+TGFβ) controls (Figure 1).

**Figure 2:**
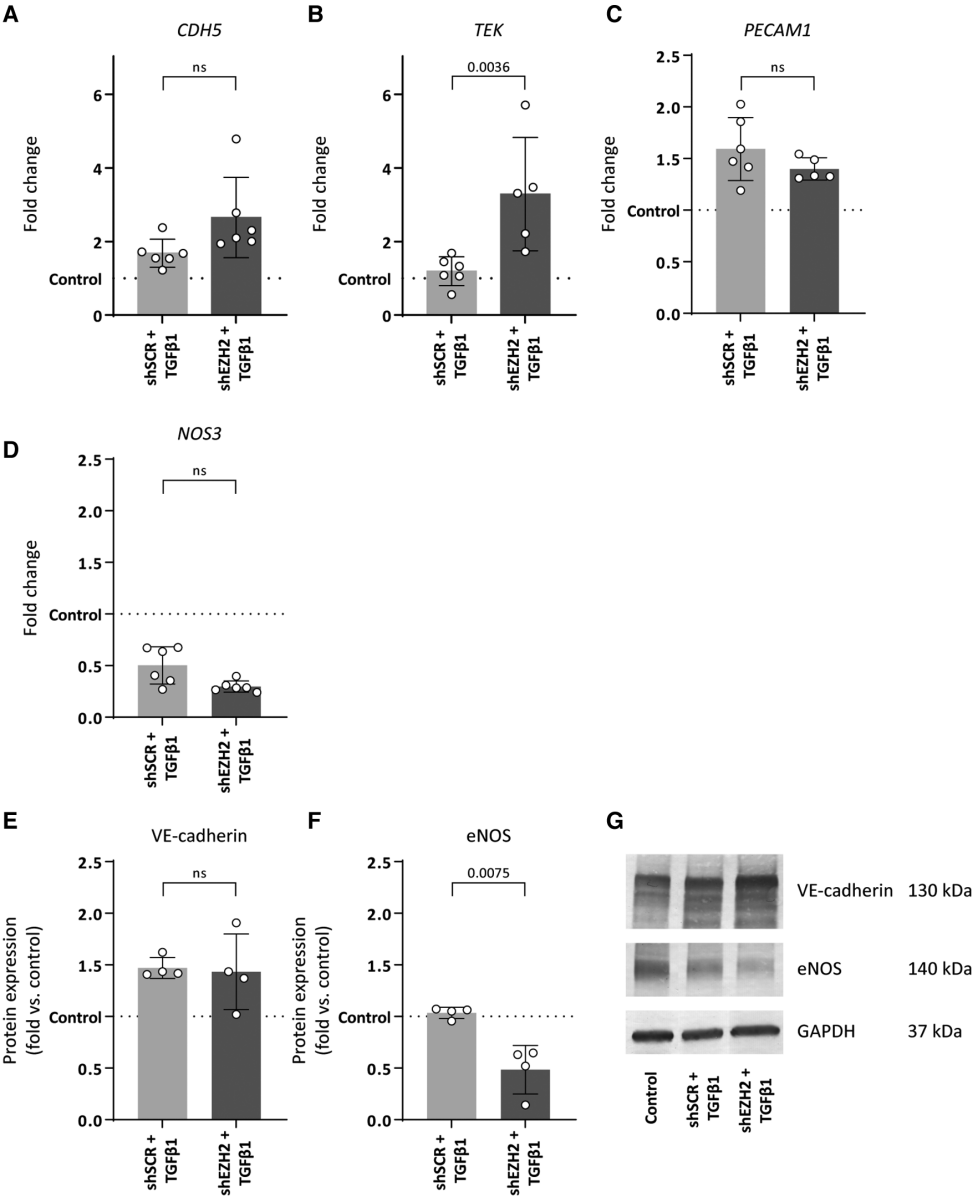
Reduction of EZH2 in TGFβ1 induced EndMT does not affect endothelial-associated gene and protein expression levels. (**A–D**) Gene expression levels of CDH5, TEK, PECAM1 and NOS3 were determined by qRT-PCR in control, shSCR + TGFβ1 and shEZH2 + TGFβ1 treated HUVECs. Gene expression data is shown as fold change and normalized to control HUVECs (*n* = 6). (**E,F**) Protein expression levels of VE-cadherin and eNOS were determined by western blot in control, shSCR + TGFβ1 and shEZH2 + TGFβ1 treated HUVECs. Protein expression data is shown as fold difference and normalized to control HUVECs (*n* = 4). (**G**) Representative western blotting images of control, shSCR + TGFβ1 and shEZH2 + TGFβ1 treated HUVECs for VE-cadherin and eNOS protein expression levels. Data are from *n* = 6 independent experiments. Data presented as mean ± SD, *P*-values from one-way ANOVA with Sidak's multiple comparison tests.

To induce EndMT, HUVEC were treated with TGFβ1 as described before (7, 31, 32). Stimulation of HUVEC with TGFβ1 decreased the expression of *NOS3* (2.0-fold, *p* = 0.0049) and increased the expression of *PECAM1* (1.6-fold, *p* = 0.011) on gene expression levels as compared to untreated control cells, while expression of *CDH5* and *TEK* remained unchanged (Figures 2A–D). On the protein level, VE-cadherin expression increased (1.5-fold, *p* = 0.0319), and the expression of eNOS remained unchanged (Figures 2E,F). The reduction in H3K27Me3 abundance did not affect endothelial gene expression, except for *TEK*, which increased (2.8-fold) compared to the shSCR controls (Figure 2B). Notably, on protein level, the decrease in eNOS expression was aggravated in shEZH2 HUVEC when compared to shSCR controls (Figures 2F,G).

**Figure 3:**
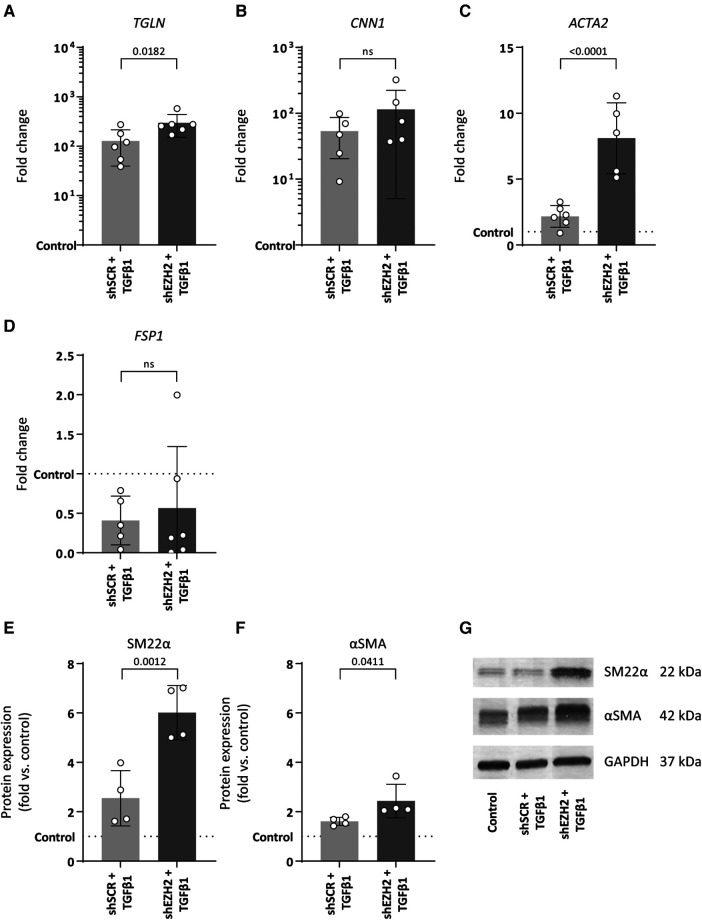
Reduction of EZH2 in TGFβ1 induced EndMT increases mesenchymal and fibrosis-associated gene and protein expression levels. (**A–D**) Gene expression levels of TGLN, CNN1, ACTA2 and FSP1 were determined by qRT-PCR in control, shSCR + TGFβ1 and shEZH2 + TGFβ1 treated HUVECs. Gene expression data is shown as fold change and normalized to control HUVECs (*n* = 6). (**E,F**) Protein expression levels of SM22α and αSMA were determined by western blot in control, shSCR + TGFβ1 and shEZH2 + TGFβ1 treated Huvecs. Protein expression data is shown as fold difference and normalized to control HUVECs (*n* = 4). (**G**) Representative western blotting images of control, shSCR + TGFβ1 and shEZH2 + TGFβ1 treated HUVECs for SM22α and αSMA protein expression levels. Data presented as mean ± SD, *P*-values from one-way ANOVA with Sidak's multiple comparison tests.

Consistent with the induction of EndMT by TGFβ1, the expression of mesenchymal genes was induced in TGFβ1 stimulated HUVEC and aggravated by the reduction of H3K27Me3 (Figures 3A–D). For example, the expression of *TAGLN* and *CNN1* increased 127.5- and 53.4-fold upon TGFβ1 stimulation (*p* = 0.0788 and *p* = 0.3414), which increased to 295.9- and 114.3-fold in shEZH2 HUVEC (Figures 3A,B). Similar, on protein level, SM22α and αSMA increased after the reduction in H3K27Me3 abundance compared to shSCR controls (Figures 3E–G).

**Figure 4:**
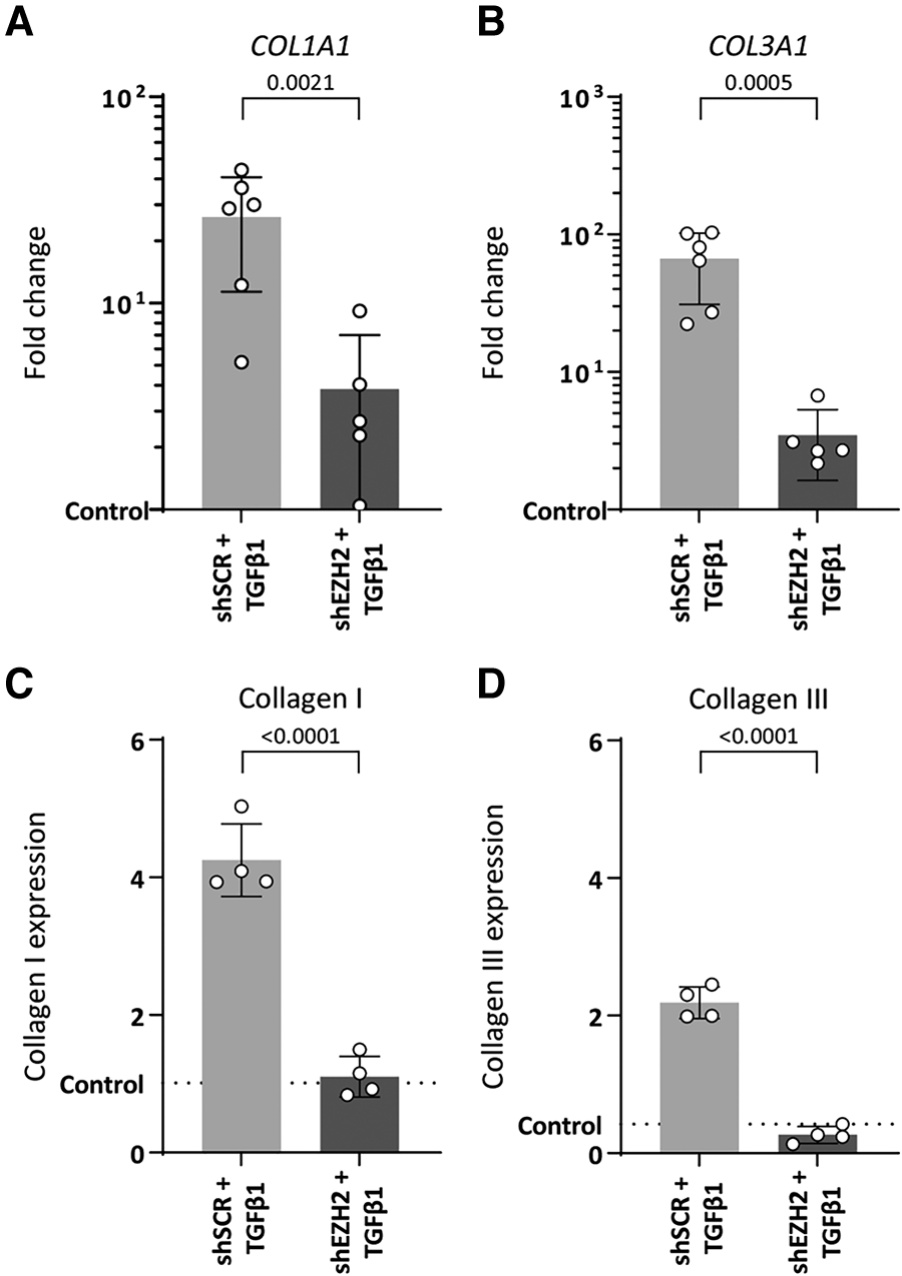
Collagen expression is reduced in H3K27Me3-deficient HUVECs. (**A,B**) Gene expression levels of COL1A1 and COL3A1 were determined by qRT-PCR in control, shSCR + TGFβ1 and shEZH2 + TGFβ1 treated HUVECs. Gene expression data is shown as fold change and normalized to control HUVECs (*n* = 6). (**C,D**) Protein expression levels of COL1A1 were determined by staining in control, shSCR + TGFβ1 and shEZH2 + TGFβ1 treated HUVECs. Protein expression data is shown as fold change and normalized against DNA content (*n* = 4). Data presented as mean ± SD, *P*-values from one-way ANOVA with Sidak's multiple comparison tests.

EndMT is associated with increased extracellular matrix production induced by TGFβ1. Indeed, TGFβ1-stimulated HUVEC increased gene expression of ECM components *COL1A1* and *COL3A1* (26.1-fold, *p* = 0.0005 and 66.6-fold, *p* = 0.0002, respectively) and protein expression of collagen I and collagen III (4.2-fold, *p* ≤ 0.0001 and 5.2-fold, *p *≤ 0.0001) compared to unstimulated control cells (Figure 4).

Counterintuitively, and in contrast to the aggravated induction of mesenchymal gene expression, lowering the H3K27Me3 abundance in TGFβ1-stimulated HUVEC blunts the expression of these ECM genes on gene (6.8-fold and 19.2-fold for *COL1A1* and *COL3A1*, respectively, Figures 4A,B) and protein levels (3.9-fold and 10.6-fold for collagen I and collagen III, respectively, Figures 4C,D) compared to shSCR controls. In combination, these data suggest that the reduction of EZH2 and its related histone mark H3K27Me3 does not halt the EndMT phenotype but mitigates the fibrogenesis that is associated with EndMT.

### 3.2 H3K27Me3 abundance affects the expression of collagen-targeting miR-29c

**Figure 5:**
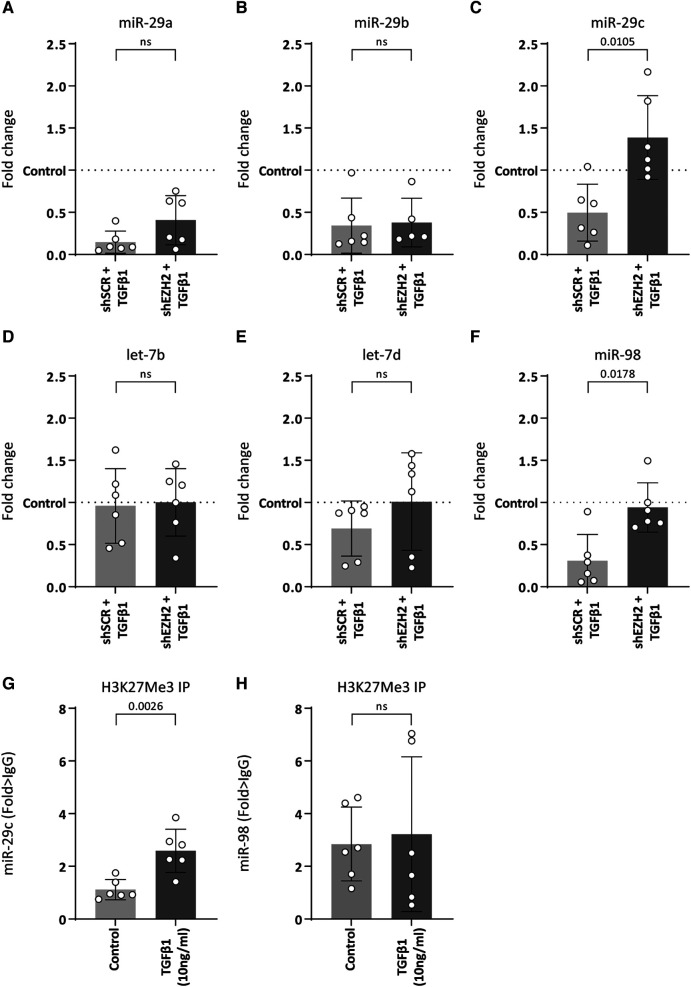
miR-29c expression is regulated by H3K27Me3 abundance. (**A–F**) Expression levels of miR-29a, miR-29b, miR-29c, let-7b, let-7d and miR-98 were determined by qRT-PCR in control, shSCR + TGFβ1 and shEZH2 + TGFβ1 treated HUVECs. Gene expression data is shown as fold change and normalized to control HUVECs (*n* = 6). (**G,H**) The abundance of DNA encoding for miR-29c and miR-98 after H3K27Me3 chromatin immunoprecipitation (ChIP) was determined in control and TGFβ1 treated HUVEC (*n* = 6). DNA abundance is shown as fold change over IgG. Data presented as mean ± SD, *P*-values from one-way ANOVA with Sidak's multiple comparison tests.

As H3K27Me3 is a transcriptional repressor, the possibility of a direct interaction between H3K27Me3 and collagen is unlikely. Rather, the co-occurring decreases suggests another layer of regulation. It is widely accepted that the expression of collagens is co-regulated by microRNAs (miRNAs) (33–36) and changes in H3K27Me3 abundance associates with changes in miRNA expression levels (37, 38). For example, studies have shown that members of the miR-29 family were significantly reduced upon TGFβ1 expression, which associates with an increase in collagen expression (33, 34). Additionally, several studies show that pharmacological or siRNA-based knockdown models of EZH2 led to an upregulation of miR-29b due to reduced H3K27Me3 abundance (37, 38). Besides the miR-29 family, a literature search showed that other miRNAs that could be involved in collagen regulation and might be affected by EZH2 are the let-7 family including let7b and let-7d and miR-98. Therefore, we hypothesized that H3K27Me3 abundance affects the expression of collagen-targeting miRNAs during EndMT. Stimulation of HUVEC with TGFβ1 decreased the expression of miR-29a (6.8-fold, *p* = 0.0014), miR-29b (2.9-fold, *p* = 0.0152), miR-29c (2.0-fold, *p* = 0.1633) and miR-98 (3.3-fold, *p* = 0.0099), whereas the expression levels of the let-7 family remained unchanged (Figures 5A–F). The decrease of miR-29c and miR-98 was abolished by the reduction in H3K27Me3 abundance, whereas the expression levels of the let-7 family and the other miR-29 family members remained unchanged (Figure 5). Notably, miR-98 expression level returned to baseline, while miR-29c expression increased to above baseline by the reduction in H3K27Me3 abundance (Figures 5C,F), suggesting that their expression is (in part) epigenetically regulated by H3K27Me3. To validate that H3K27Me3 abundance is a determinant of miR-29c and miR-98 transcription, we performed chromatin immunoprecipitation of H3K27Me3. Detectable levels of DNA encoding for miR-29c, and miR-98 were present in all H3K27Me3 ChIP samples. After TGFβ1 stimulation, the abundance of DNA encoding for miR-29c increased in HUVEC, whereas no differences in the abundance of DNA encoding for miR-98 were observed. These data imply that miR-29c expression is regulated in part by H3K27Me3 abundance (Figures 5G,H).

**Figure 6:**
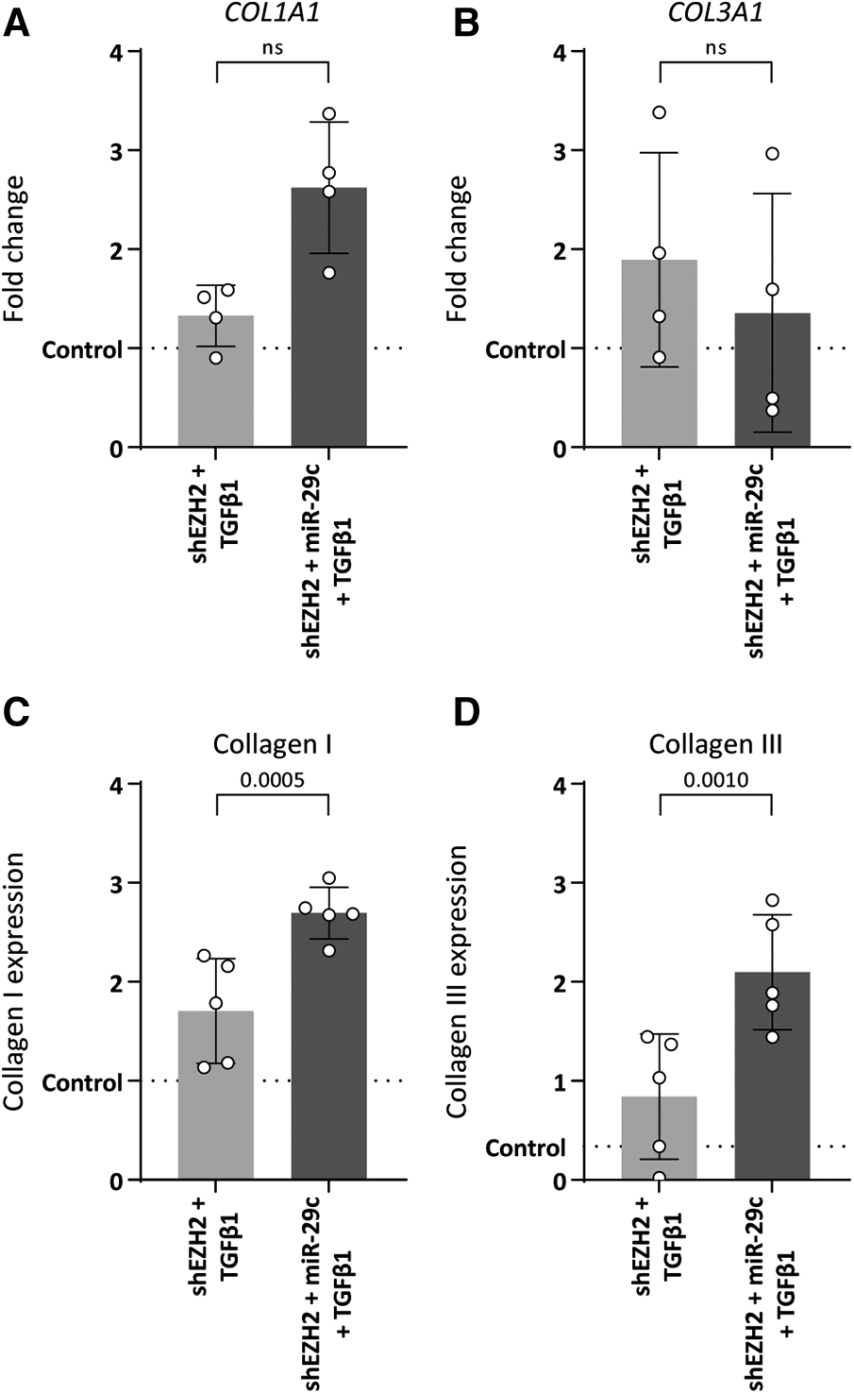
Double knockdown of EZH2 and miR-29c increases collagen expression. HUVECs were stably transduced with a short hairpin constructed directed against EZH2 (shEZH2) or with a non-targeting control (shSCR), followed by transfection with a siRNA construct against miR-29c and stimulation with 10 ng/ml TGFβ1 for 72 h. (**A,B**) Gene expression levels of COL1A1 and COL3A1 were determined by qRT-PCR in control, shEZH2 + TGFβ1 and shEZH2 + miR-29c + TGFβ1 treated HUVECs. Gene expression data is shown as fold change and normalized to control HUVECs (*n* = 4). (**C,D**) Protein expression levels of COL1A1 were determined by staining in control, shSCR + TGFβ1 and shEZH2 + TGFβ1 treated HUVECs. Protein expression data is shown as fold change and normalized against DNA content (*n* = 5). Data presented as mean ± SD, *P*-values from one-way ANOVA with Sidak's multiple comparison tests.

MiR-29c reportedly modulates collagen expression in ECs (33). Indeed, the depletion of miR-29c in HUVEC with low H3K27Me3 abundance restored collagen expression without affecting mesenchymal gene expression, except for *ACTA2* which increased 2.0-fold as compared to H3K27Me3-deficient HUVEC (Supplementary Figure S2). Specifically, the stimulation of shSCR HUVEC with TGFβ1 increased the expression of *COL1A1* and *COL3A1* on gene (5.4-fold, *p* = 0.0011 and 6.0-fold, *p* = 0.0017, respectively) and protein levels (3.7-fold, *p *≤ 0.0001 and 9.4-fold, *p *≤ 0.0001 fold respectively) (data not shown). This increase was blunted in H3K27Me3-deficient HUVEC (Figure 6), whereas the simultaneous blunting of miR-29c expression partly restored *COL1A1* expression again by 2.0-fold (gene expression), although this failed to reach significance and collagen I expression by 1.6-fold and collagen III expression by 2.5-fold compared to HUVEC only deficient in H3K27Me3 (Figures 6A–D). These data imply that collagen I and collagen III expression is regulated by miR-29c during TGFβ1 induced EndMT.

### 3.3 H3K27Me3 abundance associates with an increased presence of perivascular fibrosis and a reduced miR-29c expression in PAH rats

**Figure 7:**
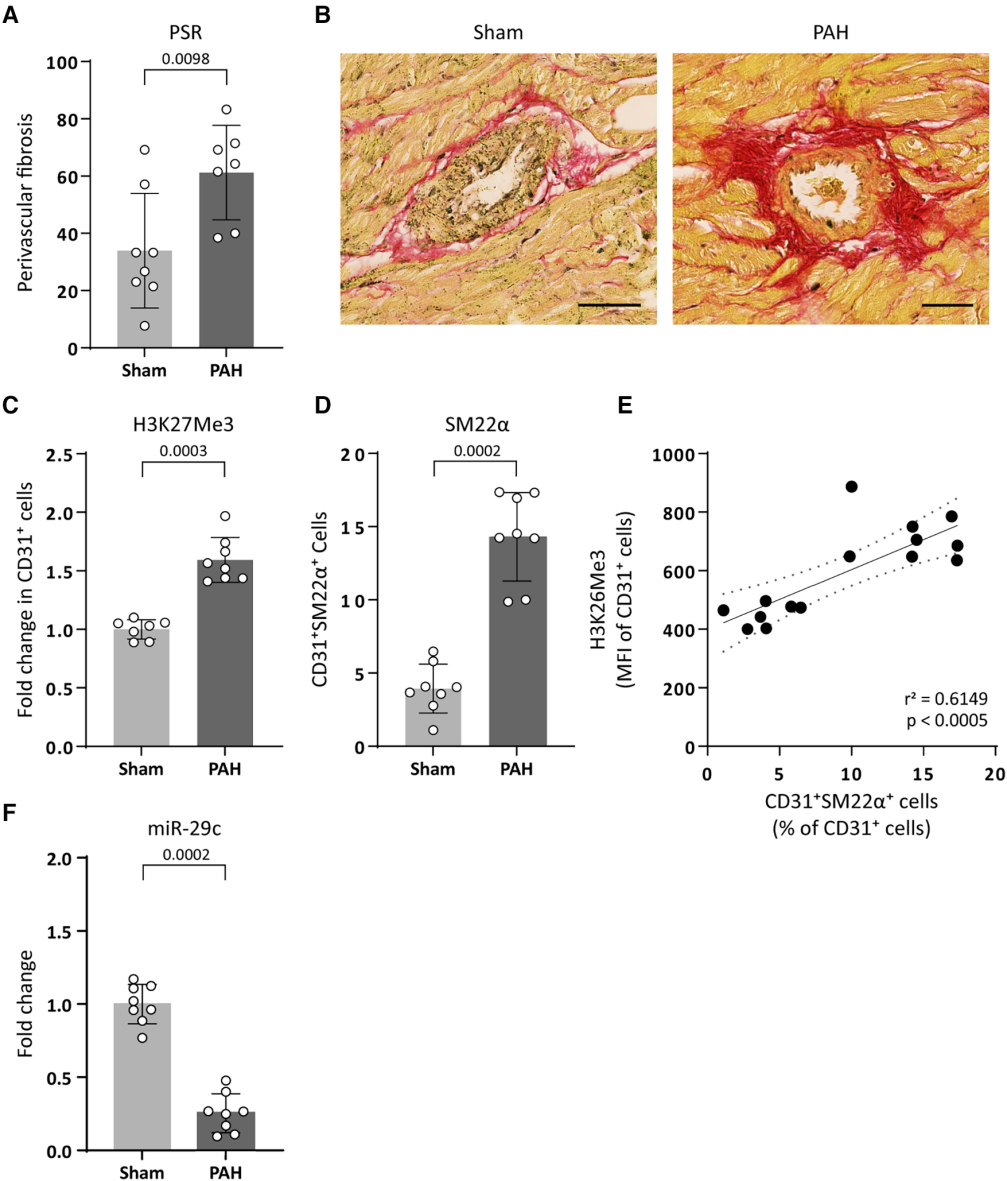
PAH associates with an increased H3K27Me3 abundance and a decreased miR-29c expression. (**A**) Fibrogenesis was determined by quantification of perivascular PSR staining around arteries in PAH and sham treated rats. Data is shown as the percentage of PSR positive arteries over total amount of arteries. (**B**) Representative images of PSR stained arteries in sham or PAH animals (sham *n* = 8, PAH *n* = 7). Scale bars represent 50 µm. (**C**) representative images of H3K27Me3 staining (sham *n* = 7, PAH *n* = 8) and (**D**) SM22α staining in CD31^+^ cells of PAH or sham treated rats (sham *n* = 8, PAH *n* = 8). Data is shown as fold change in CD31^+^ cells (**C**) or as percentage of SM22α^+^/CD31^+^ cells (**D**) and normalized to sham treated rats. (**E**) H3K27Me3 abundance is positively correlated with SM22α^+^ expression levels in CD31^+^ cells. Correlation was analysed performing Pearson correlation test (*r* = 0.7842, *p* = 0.0005) followed by simple linear regression (**F**) miR-29c expression levels were determined by RT-qPCR in PAH or sham treated rats. Data is shown as fold change and normalized to sham treated rats (sham *n* = 8, PAH *n* = 8). Data presented as mean ± SD, *P*-values from Mann-Whitney test.

To translate our findings to CVD pathology, we investigated if increased H3K27Me3 abundance affects the expression of the collagen-targeting miR-29c *in vivo* using an EndMT-prone model of pulmonary arterial hypertension (PAH). PAH is a disease characterized by increased right ventricular fibrosis consisting predominantly of interstitial and perivascular fibrosis (10, 39, 40). In this model, the development of PAH is observed as an increased mean pulmonary arterial pressure (mPAP) in PAH animals (31.17 ± 7.31, *p* = 0.0130), compared to sham control animals (21.60 ± 2.51) and an increased pulmonary vascular resistance (PVR, 0.303 ± 0.156 PAH and 0.083 ± 0.017 sham, *p* = 0.0159). As a consequence of increased mPAP and PVR, the right ventricle (RV) adapts to the increased afterload, eventually resulting in fibrosis (remodelling) and RV failure as indicated by increased RV weight (418.3 ± 99.89 PAH and 195.0 ± 22.61 sham, *p* = 0.0022 respectively) and decreased RV output (110.0 ± 44.22 PAH and 176.3 ± 32.26 sham, *p* = 0.0111) (Table 3). As the onset of cardiac fibrosis during PAH is predominantly perivascular (10, 39, 40), we investigated EndMT in the perivascular areas of the right ventricle. Indeed, in PAH rats an increase in the occurrence of perivascular fibrosis is observed as compared to its occurrence in sham control animals (61% and 34%, respectively, Table 3, Figures 7A,B). Interestingly, in the perivascular areas, H3K27Me3 abundance increased 1.6-fold in ECs of PAH rats compared to sham-treated rats (Figures 7C,D) which associated with an increased percentage of CD31^+^ SM22α^+^ cells (14.31% PAH and 3.94% sham, *p* = 0.0002, Figures 7C–E), indicative of EndMT. Additionally, increased H3K27Me3 abundance associated with a decreased expression of miR-29c in PAH rats compared to sham-treated rats (3.9-fold, Figure 7F).

**Table 3:** Animal data during PAH development.

	Sham	PAH
	(*n* = 8)	(*n* = 7)
Body weight	358.0 ± 17.53	318.7 ± 16.55**
Pulmonary artery pressures (mmHg)		
Mean	21.60 ± 2.51	31.17 ± 7.31*
Systolic	27.00 ± 1.58	38.67 ± 10.67
Diastolic	14.80 ± 3.27	23.83 ± 7.08*
Heart weight (BW corrected)		
Heart weight (mg.g^−1^)	940.3 ± 75.56	1,415 ± 200.0**
RV weight (mg.g^−1^)	195.0 ± 22.61	418.3 ± 99.89**
RV weight (mg.g^−1^ BW)	0.546 ± 0.049	1.146 ± 0.139***
Right ventricular pressures (mmHg)		
Mean	12.67 ± 2.42	25.88 ± 6.94***
sRVP	20.17 ± 3.92	39.63 ± 11.84**
dRVP	6.67 ± 2.25	15.75 ± 6.78**
Pulmonary vascular resistance (mmHg.ml.min-1)	0.083 ± 0.017	0.303 ± 0.156*
Heart rate (bpm)	381.4 ± 19.54	284.0 ± 32.47***
Stroke volume (µl)	462.0 ± 92.48	378.1 ± 121.9
Cardiac output (ml/min)	176.3 ± 32.26	110.0 ± 44.22*

Continuous data are expressed as mean ± S.D. and statistical analysis was performed using the Mann-Whitney tests.

* *p* < 0.05.

** *p* < 0.01.

*** *p* < 0.001 vs. sham.

These data imply that an increased abundance of H3K27Me3 is associated with a decreased expression of miR-29c and an increase in perivascular fibrosis *in vivo*.

## 4 Discussion

In this study, we demonstrate that an increased H3K27Me3 abundance in ECs contributes to fibrogenesis in a miR-29c-dependent manner. This conclusion is supported by our observations that, upon reduction of H3K27Me3, TGFβ1-induced collagen expression was mitigated, H3K27Me3 abundance directly regulated miR-29c expression levels, and that depletion of miR-29c in ECs that underwent EndMT with low H3K27Me3 abundance restored collagen expression. Expectedly, in rats with pulmonary arterial hypertension, an increased abundance of H3K27Me3 associates with a decreased expression of miR-29c and an increase in perivascular fibrogenesis.

An increased abundance of H3K27Me3 is observed in several CVDs that associate with EndMT and fibrogenesis (23). Hence, we hypothesized that endothelial H3K27Me3 abundance may affect EndMT. Interestingly and contradictory to our hypothesis, we uncovered an inverse relation between endothelial H3K27Me3 abundance and EndMT, wherein low H3K27Me3 abundance in ECs that underwent EndMT coincided with high mesenchymal marker expression. Indeed, endothelial H3K27Me3 abundance has previously been associated with EC activation, inflammation and EndMT (18, 21). Notably, and in sharp contrast to the increased expression of mesenchymal cell markers, the EndMT-induced expression of extracellular matrix proteins was blunted by the reduction in H3K27Me3 abundance, suggesting that although a low H3K27Me3 abundance does not halt EndMT, low H3K27Me3 abundance does impede EndMT-associated fibrogenesis.

Previous reports utilizing pharmacological agents to reduce H3K27Me3 abundance *in vivo* suggest its involvement in the regulation of fibrogenesis (41–43). Specifically, systemic administration of EZH2 inhibitors (lowering H3K27Me3 abundance) associates with a decreased expression of extracellular matrix genes, including collagens and fibronectin (41–43). In contrast to our findings, in these studies, a reduced expression of extracellular matrix genes was accompanied by a decreased expression of mesenchymal genes and possible EndMT. This discrepancy may originate from discrepancies between our *in vitro* EC model and the *in vivo* pathophysiological processes. *In vitro*, we investigated the effects of reduced H3K27Me3 abundance in isolated ECs, whereas the *in vivo* systemic administration of EZH2 inhibitors may affect multiple cell types that can affect the EndMT process. For example, we previously showed that inflammatory activation of ECs drive EZH2 expression and synergizes with EndMT (4), whereas others reported that the pharmacological or genetic inhibition of EZH2 in macrophages mitigates their inflammatory signalling (44–46). In combination, it is plausible that a systemic reduction of EZH2 therefore affects both the fibrogenic processes in ECs directly and the processes underlying EndMT via indirect mechanisms, resulting in an augmented benefit of EZH2 inhibition *in vivo*. Nonetheless, the inhibition of EZH2 and its concurrent reduction in H3K27Me3 abundance in ECs results in reduced fibrogenesis.

EZH2 and its epigenetic mark H3K27Me3 are known to repress gene expression. Counterintuitively, the reduced expression of extracellular matrix genes associates with a reduced H3K27Me3 abundance, suggestive of an indirect regulatory mechanism. Of note, EZH2 is known to regulate the expression of miRNAs (32) that may affect fibrogenesis (33, 34, 47). Several miRNAs have been implicated in the regulation of fibrogenesis and although their potential regulation by EZH2 remains to be fully elucidated. We now show that the expression of the collagen-targeting miR-29c is directly regulated by H3K27Me3 abundance. Albeit our results suggest a prominent role for miR-29c in the regulation of fibrogenesis, we emphasize that endothelial fibrogenesis is not solely regulated by miR-29c. In our studies, we used a biased selection of miRNAs that are reported to regulate collagen expression. Moreover, our data shows that the depletion of miR-29c in ECs with low H3K27Me3 abundance does not fully restore collagen expression, suggesting that alternative inhibitory molecules are present. Additionally, a growing body of evidence indicates that EZH2 can directly methylate non-histone proteins independent of H3K27Me3, although these non-histone substrates are mostly transcription factors and chromatin associated proteins (48). Also, prediction of potential target genes of miR-29c using a target scan database shows an enormous amount of predicted target genes (49). We do not investigate all extracellular matrix proteins, but rather focus on collagen I and III, which could serve as an example for how EZH2 targets miR-29c and is thereby involved in the regulation of extracellular matrix proteins. Nevertheless, we provide proof-of-concept that the epigenetic regulation of miRNA expression may be an important mechanism in the regulation of fibrogenesis, which might be therapeutically exploited.

The processes of EndMT and fibrogenesis are commonly thought to coincide. Yet, our data implies that EndMT and fibrogenesis are differently regulated at the epigenetic level in ECs. Of particular interest, Tombor et al. recently showed that, in the context of acute myocardial infarction, partial EndMT (i.e., ECs that transiently adopt a mesenchymal phenotype) facilitates repair and regeneration and occurs in the absence of fibrogenesis (50). Although the regulatory mechanisms underlying this partial EndMT remained elusive, it is tempting to speculate that H3K27Me3 abundance is involved in its regulation. Specifically, low levels of H3K27Me3 may prevent ECs from transitioning from partial EndMT (i.e., regenerative EndMT) to a complete EndMT (i.e., fibrogenic EndMT). In this context, it is interesting to explore if EZH2 activity can be therapeutically modulated to inhibit complete EndMT, while maintaining regenerative or partial EndMT.

In conclusion, here we show an epigenetic regulatory pathway underlying endothelial fibrogenesis and we demonstrate that a decreased H3K27Me3 abundance in ECs blunts fibrogenesis in part via a miR-29c dependent manner. Therefore, systemic, or local reduction of H3K27Me3 could serve as a novel therapeutical approach to reduce fibrogenesis and may prove to be beneficial in fibrogenic diseases such as atherosclerosis, cardiac fibrosis, and PAH. Interestingly, such epigenetic drugs are already under development, which may warrant rapid clinical translation (51).

## Data Availability

The raw data supporting the conclusions of this article will be made available by the authors, without undue reservation.

## References

[1] Medici D Potenta S Kalluri R. Transforming growth factor-Β2 promotes snail-mediated endothelial-mesenchymal transition through convergence of Smad-dependent and Smad-independent signalling. Biochem J. (2011) 437(3):515–20. 10.1042/bj20101500 21585337 PMC4457510

[2] Díez M Musri MM Ferrer E Barberà JA Peinado VI. Endothelial progenitor cells undergo an endothelial-to-mesenchymal transition-like process mediated by Tgfbetari. Cardiovasc Res. (2010) 88(3):502–11. 10.1093/cvr/cvq236 20631156

[3] Liu RM Gaston Pravia KA. Oxidative stress and glutathione in Tgf-beta-mediated fibrogenesis. Free Radic Biol Med. (2010) 48(1):1–15. 10.1016/j.freeradbiomed.2009.09.026 19800967 PMC2818240

[4] Maleszewska M Moonen JR Huijkman N van de Sluis B Krenning G Harmsen MC. Il-1β and Tgfβ2 synergistically induce endothelial to mesenchymal transition in an nfκb-dependent manner. Immunobiology. (2013) 218(4):443–54. 10.1016/j.imbio.2012.05.026 22739237

[5] Krenning G Moonen JR van Luyn MJ Harmsen MC. Vascular smooth muscle cells for use in vascular tissue engineering obtained by endothelial-to-mesenchymal transdifferentiation (ENMT) on collagen matrices. Biomaterials. (2008) 29(27):3703–11. 10.1016/j.biomaterials.2008.05.034 18556062

[6] Moonen JR Krenning G Brinker MG Koerts JA van Luyn MJ Harmsen MC. Endothelial progenitor cells give rise to pro-angiogenic smooth muscle-like progeny. Cardiovasc Res. (2010) 86(3):506–15. 10.1093/cvr/cvq012 20083576

[7] Moonen JR Lee ES Schmidt M Maleszewska M Koerts JA Brouwer LA Endothelial-to-mesenchymal transition contributes to fibro-proliferative vascular disease and is modulated by fluid shear stress. Cardiovasc Res. (2015) 108(3):377–86. 10.1093/cvr/cvv175 26084310

[8] Evrard SM Lecce L Michelis KC Nomura-Kitabayashi A Pandey G Purushothaman KR Endothelial to mesenchymal transition is common in atherosclerotic lesions and is associated with plaque instability. Nat Commun. (2016) 7:11853. 10.1038/ncomms11853 27340017 PMC4931033

[9] Zeisberg EM Tarnavski O Zeisberg M Dorfman AL McMullen JR Gustafsson E Endothelial-to-mesenchymal transition contributes to cardiac fibrosis. Nat Med. (2007) 13(8):952–61. 10.1038/nm1613 17660828

[10] Good RB Gilbane AJ Trinder SL Denton CP Coghlan G Abraham DJ Endothelial to mesenchymal transition contributes to endothelial dysfunction in pulmonary arterial hypertension. Am J Pathol. (2015) 185(7):1850–8. 10.1016/j.ajpath.2015.03.019 25956031

[11] Mahmoud MM Serbanovic-Canic J Feng S Souilhol C Xing R Hsiao S Shear stress induces endothelial-to-mesenchymal transition via the transcription factor snail. Sci Rep. (2017) 7(1):3375. 10.1038/s41598-017-03532-z 28611395 PMC5469771

[12] Zeisberg EM Potenta SE Sugimoto H Zeisberg M Kalluri R. Fibroblasts in kidney fibrosis emerge via endothelial-to-mesenchymal transition. J Am Soc Nephrol. (2008) 19(12):2282–7. 10.1681/asn.2008050513 18987304 PMC2588112

[13] Hashimoto N Phan SH Imaizumi K Matsuo M Nakashima H Kawabe T Endothelial-mesenchymal transition in bleomycin-induced pulmonary fibrosis. Am J Respir Cell Mol Biol. (2010) 43(2):161–72. 10.1165/rcmb.2009-0031OC 19767450 PMC2937229

[14] Chen PY Qin L Li G Wang Z Dahlman JE Malagon-Lopez J Endothelial Tgf-Β signalling drives vascular inflammation and atherosclerosis. Nat Metab. (2019) 1(9):912–26. 10.1038/s42255-019-0102-3 31572976 PMC6767930

[15] Hulshoff MS Xu X Krenning G Zeisberg EM. Epigenetic regulation of endothelial-to-mesenchymal transition in chronic heart disease. Arterioscler Thromb Vasc Biol. (2018) 38(9):1986–96. 10.1161/atvbaha.118.311276 30354260

[16] Soler-Botija C Gálvez-Montón C Bayés-Genís A. Epigenetic biomarkers in cardiovascular diseases. Front Genet. (2019) 10:950. 10.3389/fgene.2019.00950 31649728 PMC6795132

[17] Prasher D Greenway SC Singh RB. The impact of epigenetics on cardiovascular disease. Biochem Cell Biol. (2020) 98(1):12–22. 10.1139/bcb-2019-0045 31112654

[18] Dreger H Ludwig A Weller A Stangl V Baumann G Meiners S Epigenetic regulation of cell adhesion and communication by Enhancer of Zeste Homolog 2 in human endothelial cells. Hypertension. (2012) 60(5):1176–83. 10.1161/hypertensionaha.112.191098 22966008

[19] Smits M Mir SE Nilsson RJ van der Stoop PM Niers JM Marquez VE Down-regulation of miR-101 in endothelial cells promotes blood vessel formation through reduced repression of Ezh2. PLoS One. (2011) 6(1):e16282. 10.1371/journal.pone.0016282 21297974 PMC3030563

[20] Mitić T Caporali A Floris I Meloni M Marchetti M Urrutia R Ezh2 modulates angiogenesis in vitro and in a mouse model of limb ischemia. Mol Ther. (2015) 23(1):32–42. 10.1038/mt.2014.163 25189741 PMC4426795

[21] Maleszewska M Vanchin B Harmsen MC Krenning G. The decrease in histone methyltransferase Ezh2 in response to fluid shear stress alters endothelial gene expression and promotes quiescence. Angiogenesis. (2016) 19(1):9–24. 10.1007/s10456-015-9485-2 26416763 PMC4700080

[22] Xu S Xu Y Yin M Zhang S Liu P Koroleva M Flow-dependent epigenetic regulation of Igfbp5 expression by H3k27Me3 contributes to endothelial anti-inflammatory effects. Theranostics. (2018) 8(11):3007–21. 10.7150/thno.21966 29896299 PMC5996356

[23] Yuan JL Yin CY Li YZ Song S Fang GJ Wang QS. Ezh2 as an epigenetic regulator of cardiovascular development and diseases. J Cardiovasc Pharmacol. (2021) 78(2):192–201. 10.1097/fjc.0000000000001062 34029268

[24] Aljubran SA Cox R Jr. Tamarapu Parthasarathy P Kollongod Ramanathan G Rajanbabu V Bao H Enhancer of Zeste Homolog 2 induces pulmonary artery smooth muscle cell proliferation. PLoS One. (2012) 7(5):e37712. 10.1371/journal.pone.0037712 22662197 PMC3360676

[25] Shi ZL Fang K Li ZH Ren DH Zhang JY Sun J. Ezh2 inhibition ameliorates transverse aortic constriction-induced pulmonary arterial hypertension in mice. Can Respir J. (2018) 2018:9174926. 10.1155/2018/9174926 29854032 PMC5960552

[26] Garcia R Diebold S. Simple, rapid, and effective method of producing aortocaval shunts in the rat. Cardiovasc Res. (1990) 24(5):430–2. 10.1093/cvr/24.5.430 2142618

[27] van Albada ME Schoemaker RG Kemna MS Cromme-Dijkhuis AH van Veghel R Berger RM. The role of increased pulmonary blood flow in pulmonary arterial hypertension. Eur Respir J. (2005) 26(3):487–93. 10.1183/09031936.05.00015405 16135733

[28] Rabinovitch M Gamble W Nadas AS Miettinen OS Reid L. Rat pulmonary circulation after chronic hypoxia: hemodynamic and structural features. Am J Physiol. (1979) 236(6):H818–27. 10.1152/ajpheart.1979.236.6.H818 443445

[29] Burgess WH Mehlman T Friesel R Johnson WV Maciag T. Multiple forms of endothelial cell growth factor. Rapid isolation and biological and chemical characterization. J Biol Chem. (1985) 260(21):11389–92. 10.1016/S0021-9258(17)39038-5 3900060

[30] Franco-Barraza J Beacham DA Amatangelo MD Cukierman E. Preparation of extracellular matrices produced by cultured and primary fibroblasts. Curr Protoc Cell Biol. (2016) 71:10.9.1–34. 10.1002/cpcb.2 27245425 PMC5058441

[31] Correia AC Moonen JR Brinker MG Krenning G. Fgf2 inhibits endothelial-mesenchymal transition through microrna-20a-mediated repression of canonical Tgf-Β signaling. J Cell Sci. (2016) 129(3):569–79. 10.1242/jcs.176248 26729221

[32] Vanchin B Sol M Gjaltema RAF Brinker M Kiers B Pereira AC Reciprocal regulation of endothelial-mesenchymal transition by Mapk7 and Ezh2 in intimal hyperplasia and coronary artery disease. Sci Rep. (2021) 11(1):17764. 10.1038/s41598-021-97127-4 34493753 PMC8423795

[33] Wang B Komers R Carew R Winbanks CE Xu B Herman-Edelstein M Suppression of microrna-29 expression by Tgf-Β1 promotes collagen expression and renal fibrosis. J Am Soc Nephrol. (2012) 23(2):252–65. 10.1681/asn.2011010055 22095944 PMC3269175

[34] van Rooij E Sutherland LB Thatcher JE DiMaio JM Naseem RH Marshall WS Dysregulation of micrornas after myocardial infarction reveals a role of miR-29 in cardiac fibrosis. Proc Natl Acad Sci U S A. (2008) 105(35):13027–32. 10.1073/pnas.0805038105 18723672 PMC2529064

[35] Cheng R Dang R Zhou Y Ding M Hua H. Microrna-98 inhibits Tgf-Β1-induced differentiation and collagen production of cardiac fibroblasts by targeting Tgfbr1. Hum Cell. (2017) 30(3):192–200. 10.1007/s13577-017-0163-0 28251559

[36] Park JT Kato M Lanting L Castro N Nam BY Wang M Repression of let-7 by transforming growth factor-Β1-induced Lin28 upregulates collagen expression in glomerular mesangial cells under diabetic conditions. Am J Physiol Renal Physiol. (2014) 307(12):F1390–403. 10.1152/ajprenal.00458.2014 25354942 PMC4269695

[37] Yin H Wang Y Wu Y Zhang X Zhang X Liu J Ezh2-mediated epigenetic silencing of miR-29/miR-30 targets Loxl4 and contributes to tumorigenesis, metastasis, and immune microenvironment remodeling in breast cancer. Theranostics. (2020) 10(19):8494–512. 10.7150/thno.44849 32754259 PMC7392008

[38] Stamato MA Juli G Romeo E Ronchetti D Arbitrio M Caracciolo D Inhibition of Ezh2 triggers the tumor suppressive miR-29b network in multiple myeloma. Oncotarget. (2017) 8(63):106527–37. 10.18632/oncotarget.22507 29290968 PMC5739753

[39] Andersen S Nielsen-Kudsk JE Vonk Noordegraaf A de Man FS. Right ventricular fibrosis. Circulation. (2019) 139(2):269–85. 10.1161/circulationaha.118.035326 30615500

[40] Ranchoux B Antigny F Rucker-Martin C Hautefort A Péchoux C Bogaard HJ Endothelial-to-mesenchymal transition in pulmonary hypertension. Circulation. (2015) 131(11):1006–18. 10.1161/circulationaha.114.008750 25593290

[41] Song S Zhang R Mo B Chen L Liu L Yu Y Ezh2 as a novel therapeutic target for atrial fibrosis and atrial fibrillation. J Mol Cell Cardiol. (2019) 135:119–33. 10.1016/j.yjmcc.2019.08.003 31408621

[42] Mimura I Hirakawa Y Kanki Y Nakaki R Suzuki Y Tanaka T Genome-wide analysis revealed that dznep reduces tubulointerstitial fibrosis via down-regulation of pro-fibrotic genes. Sci Rep. (2018) 8(1):3779. 10.1038/s41598-018-22180-5 29491489 PMC5830881

[43] Xiao X Senavirathna LK Gou X Huang C Liang Y Liu L. Ezh2 enhances the differentiation of fibroblasts into myofibroblasts in idiopathic pulmonary fibrosis. Physiol Rep. (2016) 4(17):e12915. 10.14814/phy2.12915 27582065 PMC5027349

[44] Zhang X Wang Y Yuan J Li N Pei S Xu J Macrophage/microglial Ezh2 facilitates autoimmune inflammation through inhibition of Socs3. J Exp Med. (2018) 215(5):1365–82. 10.1084/jem.20171417 29626115 PMC5940261

[45] Neele AE Chen HJ Gijbels MJJ van der Velden S Hoeksema MA Boshuizen MCS Myeloid Ezh2 deficiency limits atherosclerosis development. Front Immunol. (2020) 11:594603. 10.3389/fimmu.2020.594603 33574814 PMC7871783

[46] Bao X Liu X Liu N Zhuang S Yang Q Ren H Inhibition of Ezh2 prevents acute respiratory distress syndrome (ards)-associated pulmonary fibrosis by regulating the macrophage polarization phenotype. Respir Res. (2021) 22(1):194. 10.1186/s12931-021-01785-x 34217280 PMC8255011

[47] Lew WY Bayna E Dalle Molle E Contu R Condorelli G Tang T. Myocardial fibrosis induced by exposure to subclinical lipopolysaccharide is associated with decreased miR-29c and enhanced Nox2 expression in mice. PLoS One. (2014) 9(9):e107556. 10.1371/journal.pone.0107556 25233448 PMC4169435

[48] Wang J Wang GG. No easy way out for Ezh2: its pleiotropic, noncanonical effects on gene regulation and cellular function. Int J Mol Sci. (2020) 21(24):107115. 10.3390/ijms21249501 PMC7765048 33327550

[49] Yang J Hu QC Wang JP Ren QQ Wang XP Luoreng ZM RNA-Seq reveals the role of miR-29c in regulating inflammation and oxidative stress of bovine mammary epithelial cells. Front Vet Sci. (2022) 9:865415. 10.3389/fvets.2022.865415 35433915 PMC9011060

[50] Tombor LS John D Glaser SF Luxán G Forte E Furtado M Single cell sequencing reveals endothelial plasticity with transient mesenchymal activation after myocardial infarction. Nat Commun. (2021) 12(1):681. 10.1038/s41467-021-20905-1 33514719 PMC7846794

[51] Fledderus J Vanchin B Rots MG Krenning G. The endothelium as a target for anti-atherogenic therapy: a focus on the epigenetic enzymes Ezh2 and Sirt1. J Pers Med. (2021) 11(2):103. 10.3390/jpm11020103 33562658 PMC7915331

