# Supplementary material for: H3K27Me3 abundance increases fibrogenesis during endothelial-to-mesenchymal transition via the silencing of microRNA-29c
Source: Front Cardiovasc Med. 2024 May 7;11:1373279. doi: 10.3389/fcvm.2024.1373279 (PMC11106376; doi:10.3389/fcvm.2024.1373279)
Supplement: Supplementary file 1 [file Datasheet1.docx]

Supplementary Material

**1. Supplementary Figures**

**1.1 Supplementary figure 1**


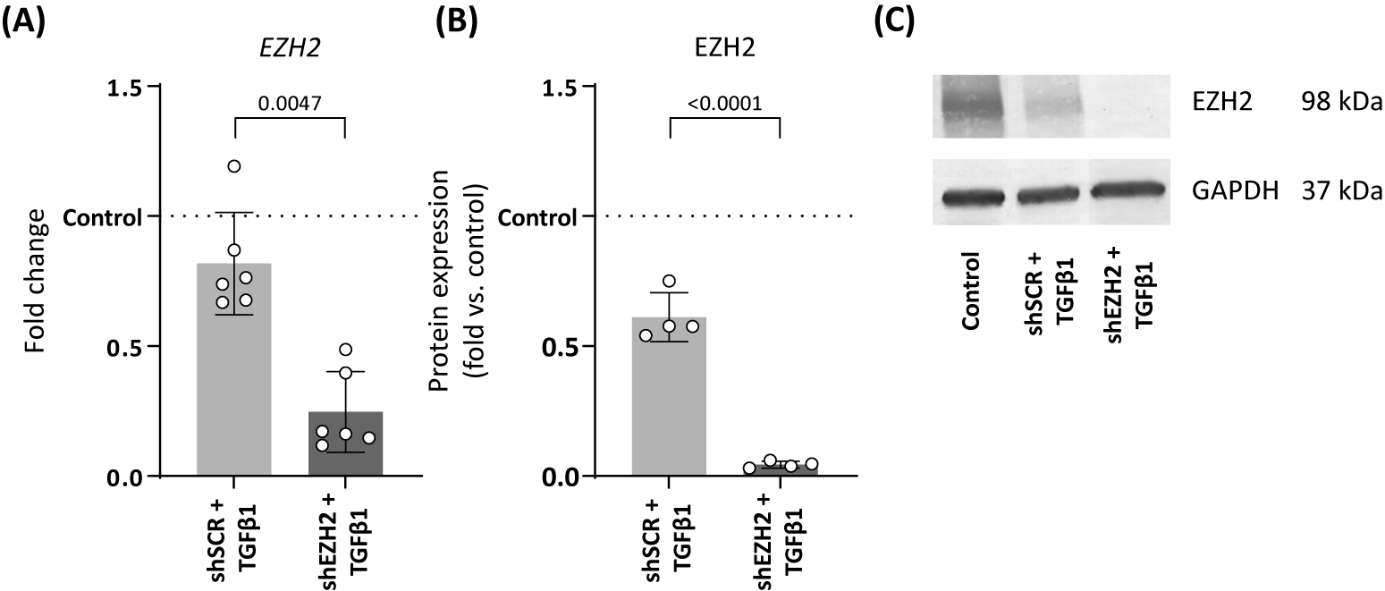


***Supplementary figure 1. EZH2 gene and protein expression is downregulated after transduction with a short hairpin against EZH2.*** HUVECs were stably transduced with a short hairpin construct directed against EZH2 (shEZH2) or with a non-targeting control (shSCR) and stimulated with 10ng/ml TGFβ1 for 72 hours. **(A)** Gene expression level of EZH2 was determined by qRT-PCR in control, shSCR + TGFβ1 and shEZH2 + TGFβ1 treated HUVECs. Gene expression data is shown as fold change and normalized to control HUVECs (n=6). **(B)** Protein expression levels of EZH2 was determined by western blot in control, shSCR + TGFβ1 and shEZH2 + TGFβ1 treated Huvecs (n=4). Protein expression data is shown as fold difference and normalized to control HUVECs. **(C)** Representative western blotting images of control, shSCR + TGFβ1 and shEZH2 + TGFβ1 treated HUVECs for EZH2 protein expression levels. Data presented as mean ± SD, P values from one-way ANOVA with Sidak’s multiple comparison tests.

**1.2 Supplementary figure 2**


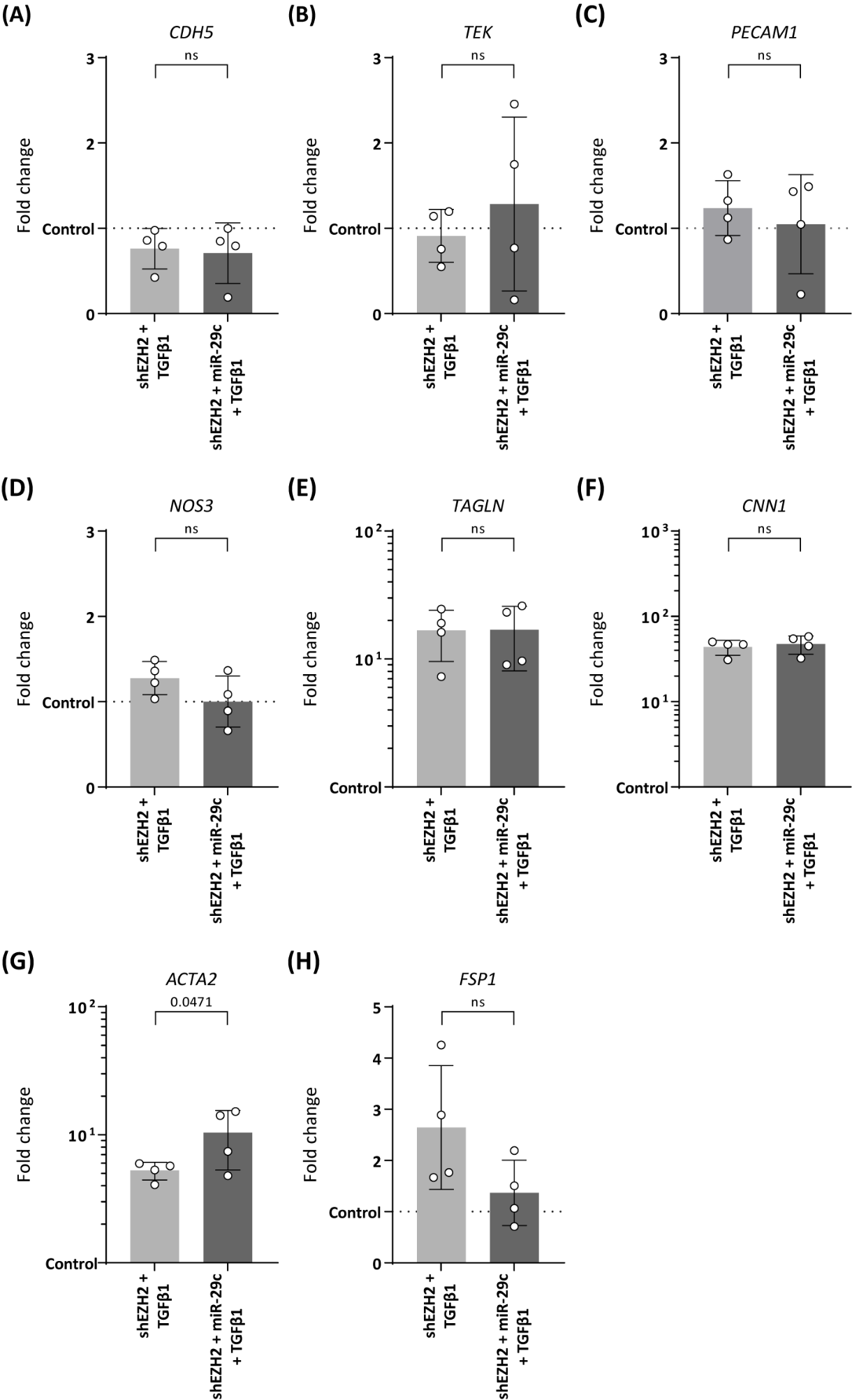


***Supplementary figure 2. Gene expression levels of EndMT associated markers in EZH2/miR-29c deficient HUVECs remain unchanged.*** HUVECs were stably transduced with a short hairpin constructed directed against EZH2 (shEZH2) or with a non-targeting control (shSCR), followed by transfection with a siRNA construct against miR-29c and stimulation with 10ng/ml TGFβ1 for 72h. **(A-H)** Gene expression levels of CDH5, TEK, PECAM1, NOS3, TGLN, CNN1, ACTA2 and FSP1 were determined by qRT-PCR in control, shEZH2 + TGFβ1 and shEZH2 + miR-29c + TGFβ1 treated HUVECs. Gene expression data is shown as fold difference and normalized to control HUVECs (n=4). Data presented as mean ± SD, P values from one-way ANOVA with Sidak’s multiple comparison tests.
